# Platypnea-orthodeoxia Syndrome in a Patient with Cryptogenic Liver Cirrhosis: An Elusive Cause of Hypoxemia

**DOI:** 10.7759/cureus.3846

**Published:** 2019-01-08

**Authors:** Edward Rojas, Adem Aktas, Hardik Parikh, Umar S Khawaja, Kathleen Pergament

**Affiliations:** 1 Internal Medicine, Rutgers New Jersey Medical School, Newark, USA

**Keywords:** hepatopulmonary syndrome, platypnea, orthodeoxia, liver, cirrhosis, methotrexate, cryptogenic

## Abstract

Platypnea-orthodeoxia syndrome (POS) has been defined as shortness of breath and hypoxemia in the upright position that improves with dorsal decubitus. This is a rare disorder caused by right-to-left shunts due to a persistent foramen ovale or pulmonary arteriovenous malformations. Hepatopulmonary syndrome can present with POS in the presence of pulmonary vasodilation and pulmonary arteriovenous communications in patients with liver disease. We report a case where the diagnosis of POS was made incidentally in a patient with cryptogenic liver cirrhosis. After other causes of hypoxemia were excluded, the diagnosis of right-to-left pulmonary shunt was confirmed by late opacification of the left heart chambers seen in a transthoracic echocardiogram. Interestingly, computerized tomography (CT) of the chest with contrast demonstrated a very prominent pulmonary vascular pattern extending to the periphery of the lungs. POS is a rare cause of hypoxemia that requires a high level of suspicion, and exclusion of more common causes of hypoxemia.

## Introduction

Platypnea-orthodeoxia syndrome (POS) describes the presence of shortness of breath and hypoxemia in the upright position with improvement of symptoms and near-normal oxygen saturation in the dorsal decubitus position [1]. POS is a rare disorder caused by right-to-left shunts due to a persistent foramen ovale (PFO) or pulmonary arteriovenous malformations. POS can be a manifestation of hepatopulmonary syndrome (HPS), defined as a triad of liver disease, inadequate oxygenation, and pulmonary vasodilation [2]. The prevalence of HPS in cirrhotic patients varies depending on various criteria used in the literature varying between 4% and 32% in some reviews, and 25.6% among liver transplant candidates [3].

Interestingly, a subgroup of clinically nonsignificant HPS has also been described. They have pulmonary vasodilation and right-to-left shunts seen on transthoracic echocardiogram (TTE) but no significant defects in oxygenation or dyspnea [2].

Mechanistically, increased pulmonary vasodilation leads to disproportionately greater perfusion without a change in ventilation (ventilation-perfusion mismatch), especially evident in the lower lobes of the lung in the upright position. This explains the phenomenon of platypnea-orthodeoxia in patients with HPS [4]. We encountered this case to be interesting for publication given that the diagnosis of POS was an incidental finding after a surgical gynecological procedure. Our patient did not present with shortness of breath, and initial anesthesiology evaluation did not reveal any contraindication for surgery.

## Case presentation

A 68-year-old woman with rheumatoid arthritis, newly-diagnosed liver cirrhosis, type 2 diabetes mellitus (T2DM), hypertension, hypothyroidism, high-grade squamous intraepithelial lesion and cervical intraepithelial neoplasia I was admitted to the obstetrics and gynecology service for a planned transvaginal hysterectomy. The patient had recently undergone an extensive workup for liver cirrhosis. There was no history of significant alcohol use and viral hepatitis serologies were negative for hepatitis A, B, and C. Ferritin level was 319 ng/ml making hemochromatosis unlikely. Anti-mitochondrial and anti-smooth muscle cell antibodies were negative. Primary biliary cirrhosis and primary sclerosing cholangitis were unlikely in the absence of other clinical findings. Wilson’s disease was also considered, however serum copper and ceruloplasmin levels were normal and the patient did not have any psychiatric symptoms. Finally, α-1 antitrypsin was within normal limits at 193 mg/dl.

Nonalcoholic fatty liver disease (NAFLD) being the leading cause of cryptogenic liver cirrhosis was found to be the most probable explanation for her cirrhosis (patient had T2DM, body mass index (BMI) was 29.83 and hyperlipidemia), nevertheless the patient had been taking methotrexate for more than 10 years for treatment of rheumatoid arthritis, and this was the second most plausible etiology in our differential.

During preoperative evaluation, the patient was noted to have a resting supine oxygen saturation of 93%. At that time, the patient denied any respiratory or cardiac symptoms, and she underwent transvaginal hysterectomy with no complications.

The patient was noted to have two grams drop in hemoglobin level after surgery which was promptly corrected with two units of packed red blood cells. On postoperative day two, the patient became dyspneic while walking to the restroom. Despite the administration of packed red blood cell transfusions, hypoxemia (oxygen saturation of 82%) sitting up and during ambulation was still noted. When the patient tried to ambulate further, she developed perioral cyanosis and increasing dyspnea. After going back to the recumbent position, her oxygenation saturation improved to 92-93%. The patient was placed on oxygen supplementation at three liters per minute by nasal cannula and her oxygen saturation remained at 93%. Internal medicine and pulmonology were consulted to further investigate the cause of her hypoxemia. On our initial evaluation, the patient was noted to have bibasilar rales and a chest X-ray revealed mild pulmonary edema. Intravenous furosemide was started which resulted in increased urine output, but no improvement of oxygen saturation.

An arterial blood gas was obtained while the patient was lying in the supine position at a fraction of inspirated oxygen of 21% showing a pH 7.49, PaCO2 30 mmHg, PaO2 53 mmHg, HCO3 22 meq/L and O2 saturation of 89.2%. The A-a gradient was 59.2 mmHg, with an expected A-a gradient for the patient’s age of 21 mmHg. This made the presumptive diagnosis of severe HPS based on the criteria by Rodriguez-Roisin et al. (Table 1) [4,5].

**Table 1:** Diagnostic criteria and severity of hepatopulmonary syndrome*. ^*^Adapted from [3]. ^**^Late opacification during contrast-enhanced transthoracic echocardiogram is defined as opacification of left-sided chambers three to six cardiac cycles after right atrium opacification.

Oxygenation defect	PaO2 < 80 mmHg and/or A-a gradient ≥15 mmHg in ambient air
Pulmonary vascular dilatation	Positive late^**^ opacification of the left heart chambers with microbubbles or abnormal uptake in the brain (>6%) with radioactive lung-perfusion scanning
Liver disease	Portal hypertension with or without cirrhosis
Severity	
Mild	A-a gradient ≥15 mmHg and PaO2 ≥ 80 mmHg
Moderate	A-a gradient ≥15 mmHg and PaO2 ≥ 60 to <80 mmHg
Severe	A-a gradient ≥15 mmHg and PaO2 ≥ 50 to <60 mmHg
Very severe	A-a gradient ≥15 mmHg and PaO2 < 50 or <300 mmHg while breathing 100% oxygen

**Figure 1:**
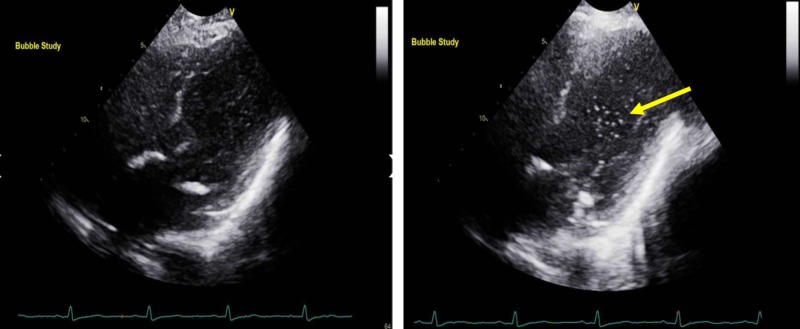
Transthoracic echocardiogram. Late opacification with microbubbles in the left heart chambers after five heart cycles are seen.

**Figure 2:**
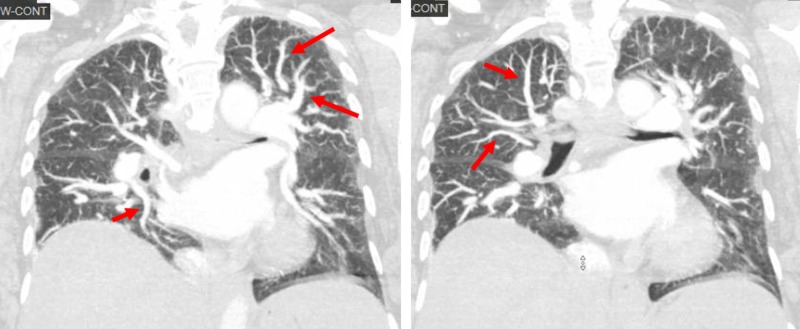
Computed tomography of the chest with intravenous contrast. Prominent pulmonary vascular dilatation extending from hilum to the periphery of the lung.

Contrast-enhanced TTE showed an ejection fraction of 55%, grade one diastolic dysfunction, and late opacification with microbubbles in the left heart chambers after five heart cycles (Figure 1). Pulmonary artery systolic pressure was 27 mm Hg. Lower extremity dopplers were negative for deep venous thrombosis (DVT) and computed tomography (CT) chest with contrast did not show pulmonary embolus (PE). Nonetheless, it did reveal a very prominent pulmonary vascular pattern extending to the periphery of the lungs (Figure 2).

Pulmonary edema was the first diagnosis in mind based on the initial chest X-ray showing pulmonary vascular congestion and rales on examination. However, her hypoxemia persisted after intravenous diuretics with an appropriate response (urine output was 4.7 liters in the next 36 hours). Other possible diagnoses were also ruled out. No signs or symptoms of pneumonia were found, her Well’s criteria showed a low probability for DVT and PE, and the patient was already on full anticoagulation with low molecular weight heparin for portal vein thrombosis, which is a common complication of advanced cirrhosis. Other causes of hypoxemia such as tense ascites and hepatic hydrothorax were also excluded.

At that point, we considered the diagnosis of interstitial lung disease. Cases of POS secondary to interstitial lung disease have been previously reported in the literature. However, the CT chest with contrast did not show findings compatible with this diagnosis [6]. Contrast-enhanced TTE demonstrated preserved ejection fraction and normal pulmonary artery systolic pressures (27 mmHg). Therefore, heart failure with reduced ejection fraction and pulmonary hypertension seemed unlikely. PFO was also ruled out given the absent early opacification of the left atrium and ventricle after agitated saline was injected. Late opacification of the left cardiac chambers made HPS secondary to intrapulmonary shunts a more probable diagnosis.

Hepatology was consulted for evaluation and to determine liver transplant eligibility. Patients with HPS and a PaO2 less than 60 mmHg (such as this case) have worse outcomes without liver transplantation and are given a higher priority than patients with other disorders [7]. Standing oxygen supplementation was started at 3 L/min by nasal cannula with improvement in subjective dyspnea and mild improvement in oxygenation. Home oxygen equipment and training were provided to the patient. Low-molecular-weight heparin for portal vein thrombosis and low-dose oral furosemide were continued.

Dyspnea improved on hospital day four, and our patient was able to ambulate without shortness of breath while using portable oxygen. Oxygen saturation standing upright also improved to 93% and remained stable. The patient was discharged in stable condition on home oxygen with follow-up appointments with hepatology and pulmonary medicine. Her MELD-Na score was 12 points. However, a diagnosis of HPS will prioritize her candidacy for possible orthotopic liver transplantation (OLT). Gastroenterology will follow up to screen for esophageal varices via upper endoscopy.

## Discussion

The first documented description of HPS was in 1884 by the German physician Flückiger, who first described a woman with liver cirrhosis, cyanosis, and digital clubbing [8]. In 1977, Kennedy and Knudson introduced the term “hepatopulmonary syndrome” in their peer-reviewed publication in Chest [9]. They described a syndrome characterized by hypoxemia aggravated by exercise, orthodeoxia, hypocapnia, and evidence of hyperdynamic circulation with otherwise normal indices of pulmonary airflow, volume, and distribution. They hypothesized that low-resistance intrapulmonary communications were likely present.

The prevalence of HPS among cirrhotic patients varies depending on different definitions and ranges from 4% to 32%. Not all patients with HPS will have POS, and some series estimate that only 25% of HPS patients would also have POS [3]. A clinically nonsignificant HPS has been described, where patients have pulmonary vasodilation and right-to-left shunt but lack any significant defects in oxygenation or dyspnea [2].

Current understanding of the pathophysiology of HPS considers an increased activity of endothelial nitric oxide (NO) synthase and inducible NO synthase in the lung, with an upregulation of NO receptors causing pulmonary vasculature vasodilatation [10]. Henceforth, the administration of intravenous methylene blue has demonstrated increased pulmonary vascular resistance and improved oxygenation by inhibition of guanylate cyclase [11]. Similarly, both almitrin bismesylate, a stimulator of carotid chemoreceptors, and indomethacin, a prostaglandin inhibitor, have shown positive but transient effects in HPS treatment [12]. These therapies are therefore not currently recommended. The only definitive treatment for HPS is OLT, but given the scarcity of available liver transplants, the most commonly prescribed treatment is home oxygen supplementation.

In a study from Dueñas et al. carried out in Barcelona, 204 candidates for OLT were followed prospectively for four years; 34.2% of the sample had HPS, and they showed that female sex was independently associated with HPS [13]. In this study, the one-year survival after OLT was not affected by the presence of HPS or liver disease severity. Schiffer et al. demonstrated that survival was significantly worse in those with a PaO2 of less than 50 mmHg [14].

Transjugular intrahepatic portosystemic shunts (TIPS) have been shown to be temporarily beneficial in managing HPS, while some other studies have shown no benefit. In a review article by Tsauo et al., they compound the results of 10 studies where portosystemic pressure gradients improved after TIPS (from 18.2 mmHg to 6.5 mmHg), with significantly improved oxygenation in 75% of patients [15]. This effect was not sustained after four months in 16.6% of patients. In 25% of patients, oxygenation remained unchanged. Conversely, Martínez-Pallí et al. showed that TIPS may not be of any benefit in managing HPS [16]. Although TIPS seemed promising in some series, most of the data were limited by small study sample size and lack of statistical power. Thus, larger prospective studies are needed to more fully evaluate the risks and benefits of TIPS in HPS.

OLT remains the definitive treatment for HPS, but the procedure itself carries significant risks for morbidity and portends a poor prognosis especially among those with decreased diffusing capacity of carbon monoxide (DLCO) on preoperative pulmonary function tests [Cheng E, Zopey R, Wang T, Busuttil R. Predictors of Poor Outcomes Following Liver Transplantation in Patients with Hepatopulmonary Syndrome. American Transplant Congress; 2013]. The most common complication is hypoxemic respiratory failure and the most common cause of death is severe sepsis with multiorgan dysfunction. Nonetheless, Deberaldini et al. compared outcomes after OLT in patients with and without HPS. They studied 59 transplant recipients (HPS group n = 25 vs. No HPS = 34) and found no significant differences in short-term and long-term survival, intensive care unit stay, and reintubation rates. Consequently, preoperative diagnosis of HPS did not seem to increase overall morbidity and mortality.

Finally, HPS with or without POS is a fascinating but complex syndrome associated with high risks of morbidity and mortality. Our current understanding of the pathophysiology of HPS had led to new therapeutic approaches which to date have yielded inconsistent results in the literature. Overall, HPS patients have poor prognoses even when there is access to OLT. Further research is required to develop methods for earlier disease detection, and interventions to prevent progression of the disease to the point of manifesting hemodynamically irreversible changes.

## Conclusions

Physicians should be able to identify possible HPS and start the initial workup to rule out other causes of hypoxemia. Obtaining a pulse oximetry and blood gas in patients with suspected HPS in both the dorsal decubitus and upright positions is of paramount importance in the initial investigation of HPS. The consideration of HPS in any liver cirrhosis patient with hypoxemia, will lead to a prompt diagnosis and early referral to hepatology. Home oxygen supplementation is a reasonable approach for HPS patients who respond favorably and are waiting for more definitive treatment with OLT.
